# Deciphering the influence of pregnancy on rheumatoid arthritis and psoriatic arthritis: insights from musculoskeletal ultrasound dynamics

**DOI:** 10.1007/s00404-025-08107-2

**Published:** 2025-07-09

**Authors:** Caroline I. Ziegler, Florian Recker, Charlotte Behning, Pia Mielczarek, Brigitte Strizek, Simon M. Petzinna, Valentin S. Schäfer

**Affiliations:** 1 Medical Clinic III for Oncology, Hematology, Immune-Oncology and Rheumatology, University Hospital Bonn, Venusberg Campus 1, 53127 Bonn, Germany; 2 Department of Obstetrics and Prenatal Medicine, Center for Obstetrics and Gynecology, University Hospital Bonn, Venusberg Campus 1, 53127 Bonn, Germany; 3 Institute for Medical Biometry, Informatics, and Epidemiology, University Hospital Bonn, Venusberg Campus 1, 53127 Bonn, Germany

**Keywords:** Autoimmune disease, Synovitis, Ultrasonography, Pregnancy, Health status indicators

## Abstract

**Objective:**

This prospective observational, hypothesis-generating study aims to investigate the impact of pregnancy on disease activity in rheumatoid arthritis (RA) and psoriatic arthritis (PsA) using musculoskeletal ultrasound (MSUS) and to explore the potential of MSUS to enhance disease monitoring during pregnancy.

**Methods:**

The study enrolled female participants divided into four groups: pregnant women with RA or PsA, non-pregnant women with RA or PsA, healthy pregnant women, and healthy non-pregnant women. Disease activity was assessed using the Rheumatoid Arthritis Impact of Disease (RAID) score and the Psoriatic Arthritis Impact of Disease (PsAID) score, as well as self-reported questionnaires on health perception. MSUS was performed using the Butterfly iQ portable ultrasound scanner, with synovial hypertrophy and joint effusion assessed via the Advanced Sonography of Large Joints in Rheumatology (aSOLAR) score.

**Results:**

A total of 105 participants were included: 15 pregnant women with RA or PsA, 30 non-pregnant women with RA or PsA, 30 healthy pregnant women, and 30 healthy non-pregnant women. In pregnant women with RA, conventional disease activity scores and the aSOLAR score showed lower values over the course of pregnancy. In contrast, non-pregnant RA patients generally presented with higher scores in both conventional assessments and the aSOLAR score. Among pregnant PsA patients, conventional scores appeared lower over time, while the aSOLAR score showed comparatively higher values in later pregnancy. Self-reported health perception was more favorable in pregnant RA and PsA patients than in their non-pregnant counterparts. The CRP levels tended to be lower in later pregnancy phases among both RA and PsA patients.

**Conclusion:**

This pioneering study demonstrates the potential impact of pregnancy on the disease activity of RA and PsA. It emphasizes the exploratory potential of MSUS as an imaging biomarker in the assessment of these conditions during pregnancy. The observed discrepancies between conventional, self-reported diagnostic tools and ultrasound findings support the hypothesis that objective imaging modalities may improve disease monitoring. Our findings warrant further validation in larger, confirmatory studies.

## What does this study add to the clinical work

**Table:** 

This study highlights the potential of MSUS as a safe, objective, and pregnancy-compatible tool to monitor disease activity in rheumatoid and psoriatic arthritis during pregnancy, revealing that conventional and self-reported measures may underestimate inflammatory activity.	

## Introduction

Rheumatoid arthritis (RA) and psoriatic arthritis (PsA) are common chronic, systemic autoimmune diseases. Up to 1.0% of people worldwide are affected by RA [1, 2], while the prevalence ratio for females is 2.5 [3, 4]. In contrary, PsA is less common, with a reported incidence of 0.1–0.5%, while being more common in female patients as well [5, 6]. PsA might present with enthesitis, dactylitis, and axial involvement [7, 8], whereas RA mainly affects the synovial membranes, causing synovitis and consequent bone erosion, destruction, and cartilage loss [9]. In both conditions, chronic pain and diminished functional capacity can result from inadequate therapy and delayed diagnosis. Musculoskeletal ultrasound (MSUS) has demonstrated efficacy in identifying inflammation and early structural joint deterioration due to its accessibility, affordability, and lack of radiation [10, 11]. Consequently, MSUS has become the primary diagnostic modality for RA and PsA. It is recommended by the European League Against Rheumatism (EULAR) for both diagnosis and monitoring of disease activity in RA patients [10]. In PsA, the Group for Research and Assessment of Psoriasis and Psoriatic Arthritis (GRAPPA) and EULAR endorse its use for detecting enthesitis and evaluating disease activity [12].

Particularly, during pregnancy, MSUS is as a safe alternative to diagnostic procedures such as magnetic resonance imaging (MRI) with gadolinium [13] or X-rays, with the latter being contraindicated due to radiation exposure to the fetus [14]. Despite its potential, the use of MSUS as a diagnostic tool in pregnant women with RA or PsA is hardly investigated, with only one case report available in the current literature [15]. This case report has described the diagnostic utility of MSUS by demonstrating its capability to visualize the course of the disease and detect active RA flare-ups during pregnancy. The recommended interval of follow-up assessments of disease activity in patients with active disease is recommended between 1 and 3 months in the German S2-guideline for patients with RA [16], who receive disease-modifying treatment. Therefore, we scheduled the study appointments every 3 months.

Reliable diagnostics for monitoring disease activity in RA and PsA during pregnancy are particularly important, as pregnancy significantly affects the course of both conditions [17, 18]. Up to 60.0% of women with RA experience clinical improvement with symptomatic relief or spontaneous remission during pregnancy [17]. However, in the postpartum period, it is very common for autoimmune diseases in general to show increased disease activity [19, 20]. In RA, 39.0–46.7% of cases experience a flare-up [21, 22]. In PsA, studies on disease activity during pregnancy present mixed results, with some studies showing clinical improvements [23–25], while others report no change or deterioration [26, 27].

The risk of autoimmune diseases to adversely affect maternal and fetal outcomes following pregnancy [28] creates a need for reliable diagnostic methods during that time. Increased disease activity during pregnancy is associated with adverse outcomes [29], such as low birth weight [30], preterm delivery, and an increased incidence of cesarean section [26, 31–34]. Early detection of disease flares is therefore critical, especially in a clinical context where therapeutic options are inherently limited due to pregnancy [35]. The ability to accurately assess and manage active disease during this period, particularly in RA, may significantly improve pregnancy outcomes for both mother and child [15].

This study is the first prospective observational investigation using handheld MSUS to evaluate disease activity in pregnant women with RA and PsA. As a hypothesis-generating approach, it aspires to improve care for pregnant women with rheumatological illnesses by providing insights on the progression of the conditions through the implementation of ultrasonography into the diagnostic regime ultimately enabling more efficient management techniques.

## Methods

### Patient characteristics

Patients diagnosed with RA or PsA, as confirmed by a board-certified rheumatologist, were prospectively enrolled from the Department of Rheumatology at the University Hospital Bonn, Germany, between February 1, 2021, and August 31, 2022. Classification criteria applied were American College of Rheumatology (ACR)/European League Against Rheumatism (EULAR) criteria of 2010 for RA [36] and Classification criteria for psoriatic arthritis (CASPAR) criteria of 2006 for PsA [37]. Participants were also required to have the necessary physical and mental capacity for study participation. Control subjects, matched for age, gender, and disease history, were recruited from the Rheumatology Department and Department of Obstetrics and Prenatal Medicine at the University Hospital Bonn, mandated to have no history of rheumatological conditions.

**Fig. 1:**
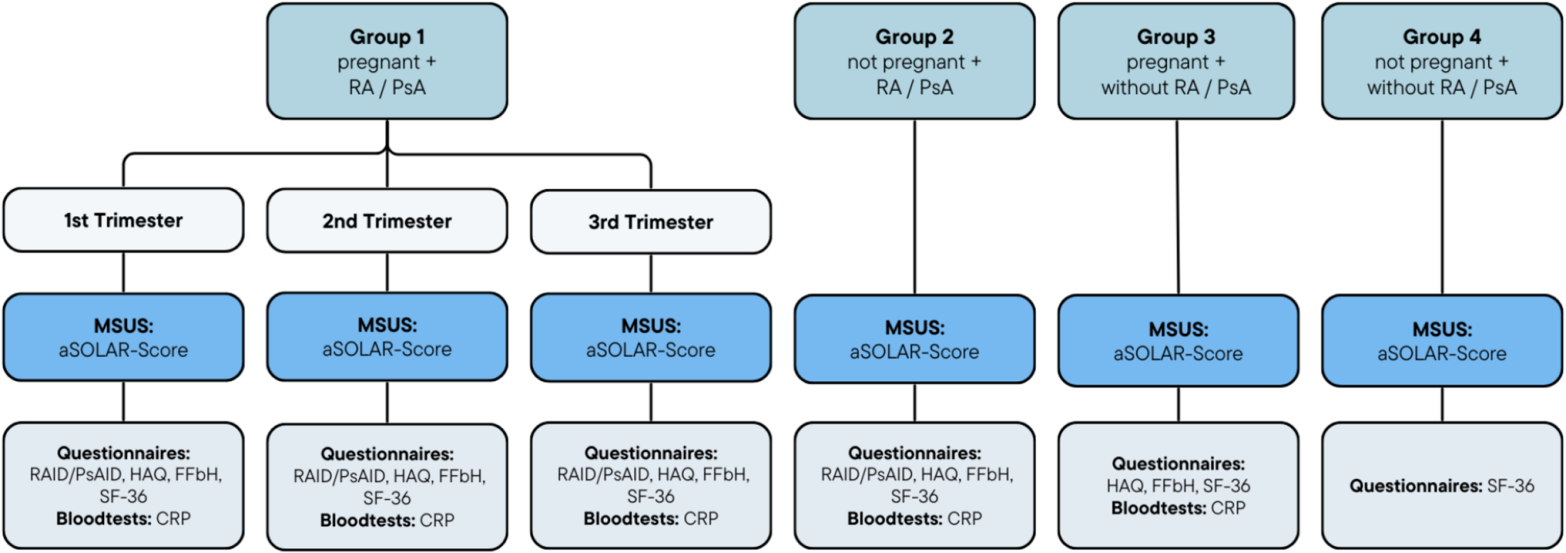
Schematic illustration of the study design. The figure outlines the study design encompassing four groups. Group 1 includes pregnant patients with RA or PsA, which were examined in each trimester of their pregnancy. Group 2 included non-pregnant patients with RA or PsA. Group 3 included healthy pregnant patients and group 4 included healthy, non-pregnant patients. Each visit included MSUS examinations and disease activity assessment with disease specific questionnaires and evaluation with CRP levels. Abbrv.: RA: rheumatoid arthritis, PsA: psoriatic arthritis, MSUS: musculoskeletal ultrasound, aSOLAR: Advanced Sonography of Large Joints in Rheumatology Score, RAID: Rheumatoid Arthritis Impact of Disease, PsAID: 12-item Psoriatic Arthritis Impact of Disease, HAQ: Health Assessment Questionnaire, FFbH: Funktionsfragebogen Hannover, SF-36: 36-Item Short Form Health Survey, CRP: C-reactive protein

The participants were categorized into four groups. The first group included pregnant women with RA or PsA. This group underwent a baseline examination in the first trimester, followed by standardized follow-up assessments in the second and third trimesters, allowing for a trimester-specific evaluation of disease activity throughout pregnancy. The second group consisted of non-pregnant women diagnosed with RA or PsA, who were examined once. The third group included healthy pregnant women, who were assessed once during the third trimester to capture physiological changes associated with advanced pregnancy. The fourth group encompassed healthy non-pregnant women, who also received a single examination. A detailed schematic overview of the study design, including all diagnostic components (functional assessments and laboratory testing), is provided in Fig. 1.

### Disease activity assessment

A comprehensive evaluation of disease activity was conducted (Fig. 1). Dependent on their diagnoses, individuals in Groups 1 and 2 were required to complete the Rheumatoid Arthritis Impact of Disease (RAID) score in case of RA [38] or the 12-item Psoriatic Arthritis Impact of Disease (PsAID) score as PsA patients [39, 40]. The 36-Item Short Form Health Survey (SF-36) [41] was administered to all groups to further assess general health perceptions. Additionally, participants in Groups 1, 2, and 3 completed the Health Assessment Questionnaire (HAQ) [42, 43] and the Funktionsfragebogen Hannover (FFbH) [44] to evaluate functional ability and health-related quality of life. Furthermore, *C*-reactive protein (CRP) levels were assessed for Groups 1, 2, and 3.

### Musculoskeletal ultrasound examination

MSUS was conducted utilizing a Butterfly iQ (Butterfly Network, Guilford, CT, USA) portable, pocket-sized ultrasound scanner [45, 46], interfaced with an Apple iPad (Apple, Cupertino, CA, USA) as the display unit. The Butterfly iQ operates within a frequency range of 1–10 MHz, provides a scan depth range of 2–30 cm, and incorporates a silicon chip equipped with a 2-D array of 9000 capacitive micro-machined ultrasound transducers. This configuration enables emulation of curved, linear, or phased transducers on-demand, supporting *M*-Mode, *B*-Mode, or Color Doppler imaging. All examinations employed the musculoskeletal preset in *B*-Mode. Prior to study start, the musculoskeletal preset and its modifications underwent testing on individuals to ensure high-quality imaging.

**Fig. 2:**
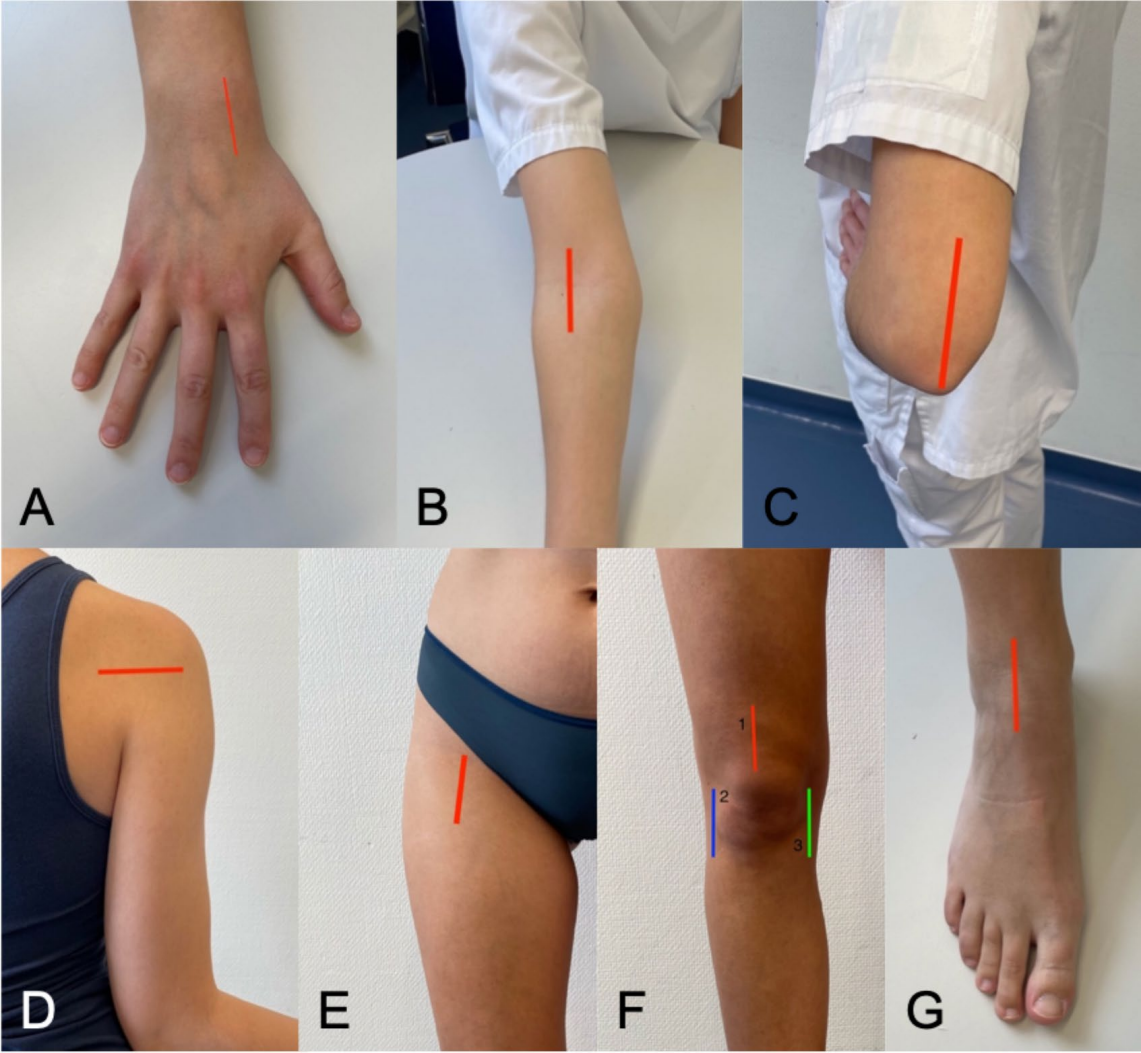
Scanning planes for the aSOLAR score. **a** Wrist: dorsal longitudinal radiocarpal section. **b** Elbow: anterior longitudinal humeroradial section. **c** Elbow: posterior longitudinal section. **d** Shoulder: posterior transversal section. **e** Hip: anterior longitudinal section. **f** Knee: 1. suprapatellar longitudinal section, 2. Lateral longitudinal section, 3. Medial longitudinal section. **g** Tibiotalar joint: anterior longitudinal section

**Fig. 3:**
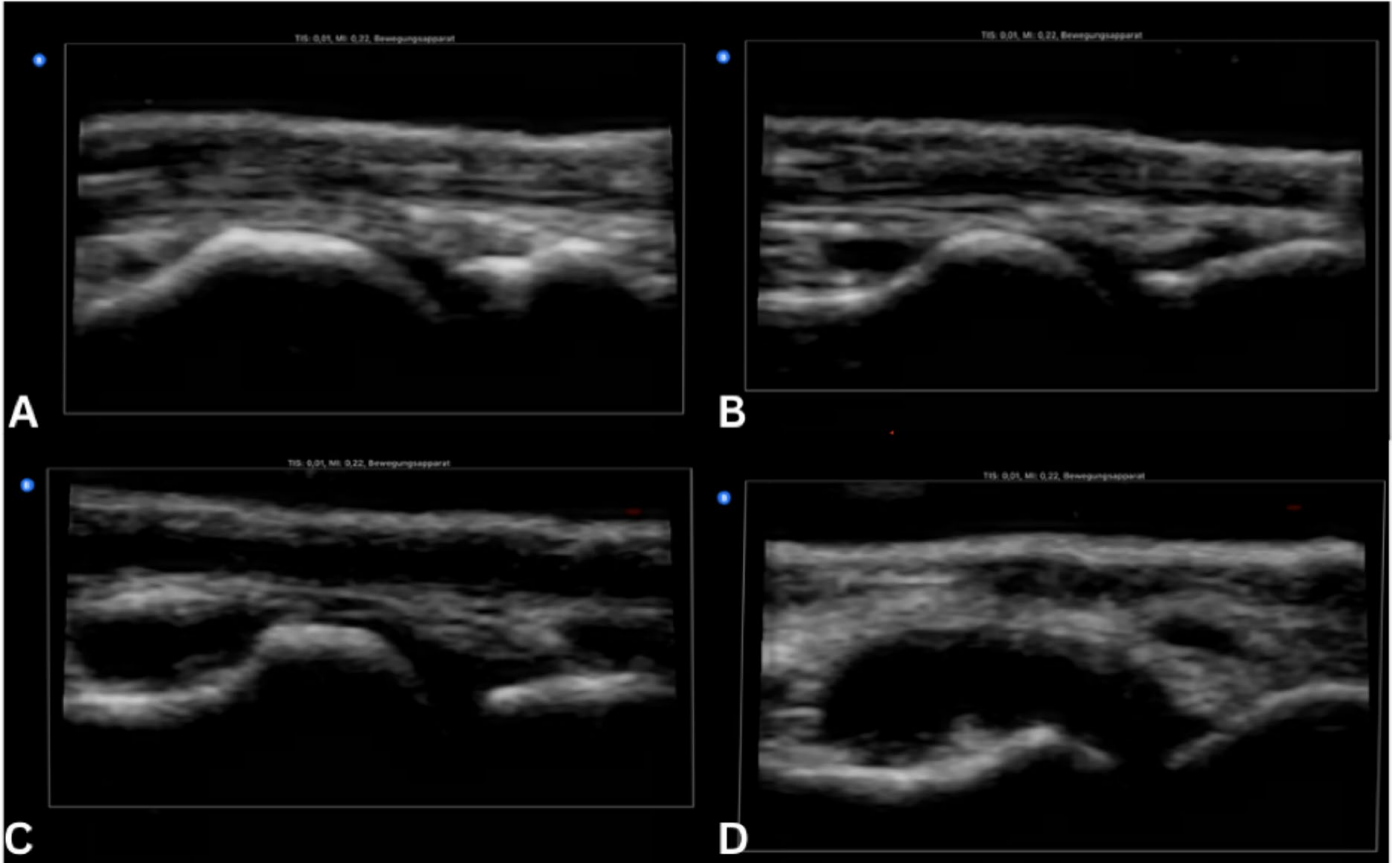
Joint synovial hypertrophy grading in the metatarsophalangeal I joint: **a** no effusion (grade 0), **b** effusion grade 1 with joint capsule distension parallel to the os metatarsals and phalanges proximales, **c** effusion grade 2 with straight joint capsule distension, and **d** effusion grade 3 with convex joint capsule distension

According to the Sonography of Large Joints in Rheumatology (SOLAR) score framework [40], nine scanning planes were used for the bilateral evaluation of seven joints as part of the MSUS scanning protocol. Additional planes were added for the wrist and tibiotalar joints as part of the Advanced Sonography of Large Joints in Rheumatology (aSOLAR) score [42] (Table 1, Fig. 2). Gray scale (GS) imaging was used in a standardized ultrasound examination regimen for each participant group in order to evaluate effusion/hyperperfusion and synovial hypertrophy. Synovial hypertrophy was assessed on a semi-quantitative scale of 0–3, corresponding to normal, mild, moderate, and severe synovitis [10]. (Fig. 3). The final aSOLAR score, which varied from 0 to 36, included quantitative evaluations of joint effusion and hyperperfusion. Notably, three scanning planes that were not evaluated in this study are included in the initial aSOLAR score; in the final computation, these were given a nominal value of zero. The scoring matrix varied according to joint type: 0–4 for the wrist, 0–8 for the elbow, 0–4 for the shoulder, 0–4 for the hip, 0–12 for the knee, and 0–4 for the tibiotalar joint. All scans were conducted by, or under the supervision of, a board-certified rheumatologist with DEGUM level III certification.

**Table 1:** Applied ultrasound planes of the aSOLAR Score: ultrasound planes were extrapolated from the Sonography of Large Joints in Rheumatology (SOLAR) score and expanded by standardized planes for the wrist and ankle joint, creating the Advanced Sonography of Large Joints in Rheumatology (aSOLAR) score

Joints	Ultrasound planes
Wrist	Dorsal longitudinal radiocarpal
Elbow	Anterior longitudinal humeroradial Posterior longitudinal
Shoulder	Posterior transverse
Hip	Anterior longitudinal
Knee	Suprapatellar longitudinal Lateral longitudinal Medial longitudinal
Tibiotalar joint	Anterior longitudinal

Every participant was examined following this ultrasound protocol

### Statistical analysis

Statistical analysis was performed using SPSS statistical software, version 29.0.2.0 (IBM, Armonk, NY, USA). Metric baseline characteristics were evaluated using mean and standard deviation (SD). The baseline characteristics included age, body mass index (BMI), CRP, aSOLAR score, and the scores of the questionnaires. Summary statistics were obtained separately for each group, trimester, and diagnosis. Changes in the metric parameters during pregnancy were evaluated graphically by displaying the mean over time. Categorical parameters, such as disease activity, were displayed in absolute and relative frequencies. All displayed results are descriptive. No statistical testing procedure was performed.

### Ethical approval

The study was conducted in accordance with the Declaration of Helsinki and was reviewed and approved by the ethics committee of the University Hospital Bonn, Germany (reference number: 406/20). Written informed consent was obtained from every patient prior to inclusion in the study.

## Results

### Patient characteristics

This study enrolled a total of 105 participants, which were categorized into four groups. Group 1 comprised 15 participants including nine patients with RA and six patients with PsA. One participant with PsA dropped out in the third trimester due to early delivery, while the others were monitored from their recruitment onwards. No drop-out occurred due to investigation associated reasons. Groups 2, 3, and 4 included 30 participants each. Detailed demographics and group assignment can be found in Table 2.

**Table 2:** Results and population characteristics: the data represent age, BMI, and results of the aSOLAR score, the HAQ-score, the RAID score or the PsAID score, the FFbH-score and the SF-36 score, as well as the CRP for each group

Group	Group 1: pregnant + RA/PsA												Group 2				Group 3		Group 4	
Diagnosis	Rheumatoid arthritis						Psoriatic arthritis						Rheumatoid arthritis		Psoriatic arthritis					
Time of survey	1st trimester		2nd trimester		3rd trimester		1st trimester		2nd trimester		3rd trimester									
Number of participants	*n* = 2		*n* = 4		*n* = 9		*n* = 1		*n* = 3		*n* = 5		*n* = 30		*n* = 30		*n* = 30		*n* = 30	
	Mean	SD	Mean	SD	Mean	SD	Mean	SD	Mean	SD	Mean	SD	Mean	SD	Mean	SD	Mean	SD	Mean	SD
Age	36	2.83	34	7.59	36	5.45	30		33	6.08	34	8.17	32	8.71	38	6.94	33	4.77	28	8.50
BMI	31.06	5.49	27.24	6.06	27.34	4.22	23.67		24.82	1.83	26.37	2.64	24.69	4.23	26.88	6.81	29.34	4.61	22.24	3.08
aSOLAR	6.50	0.71	2.50	1.73	4.33	1.87	1.00		5.33	1.53	5.80	3.27	5.33	2.50	5.25	2.57	3.52	2.31	3.30	1.86
HAQ	0.88	1.24	0.78	0.72	0.82	0.46	0.88		0.38	0.54	0.33	0.30	0.60	0.84	0.61	0.65	0.39	0.43		
RAID/PsAID	3.96	0.17	2.79	2.54	3.08	2.30	2.65		2.77	1.38	1.52	1.37	4.14	2.73	2.87	2.07				
FFbH	30.00	12.73	25.75	9.74	27.33	5.83	22.00		21.67	6.35	21.80	4.21	25.07	9.65	24.63	6.87	24.17	6.46		
SF-36	61.75	16.44	59.78	7.11	63.09	18.15	51.75		52.34	5.37	77.60	12.69	56.48	30.63	61.29	23.30	66.17	15.57	83.06	14.13
CRP	5.07	5.58	4.29	2.81	3.63	2.17	4.86		3.31	2.35	1.37	1.56	2.48	3.26	3.42	3.08	6.20	7.04		

Data are shown as mean ± SD [min; max]. Abbrv.: aSOLAR: Advanced Sonography of Large Joints in Rheumatology Score, RAID: Rheumatoid Arthritis Impact of Disease, PsAID: 12-item Psoriatic Arthritis Impact of Disease, HAQ: Health Assessment Questionnaire, FFbH: Funktionsfragebogen Hannover, SF-36: 36-Item Short Form Health Survey, CRP: *C*-reactive protein, SD: standard deviation

### Disease activity assessments

The assessment of disease activity in RA Patients showed lower RAID scores for pregnant RA patients toward the end of pregnancy, reaching 3.96 (SD ± 0.17) in the first trimester and 3.08 (SD ± 2.30) in the third. Compared to non-pregnant RA patients in Group 2, who had a mean RAID score of 4.14 (SD ± 2.73), indicating “high” disease activity, pregnant women with RA (Group 1) had a lower mean score. The assessment of disease activity in PsA Patients showed lower PsAID scores for pregnant PsA patients over time, from 2.65 in the first trimester to 1.52 (SD ± 1.32) in the third, indicating “low” disease activity throughout pregnancy. Compared to non-pregnant PsA patients in Group 2, who reached a score of 2.87 (SD ± 2.07), these scores were consistently lower. Both RA and PsA patients showed declining CRP levels during pregnancy, which was in line with the RAID and PsAID-score results.

**Fig. 4:**
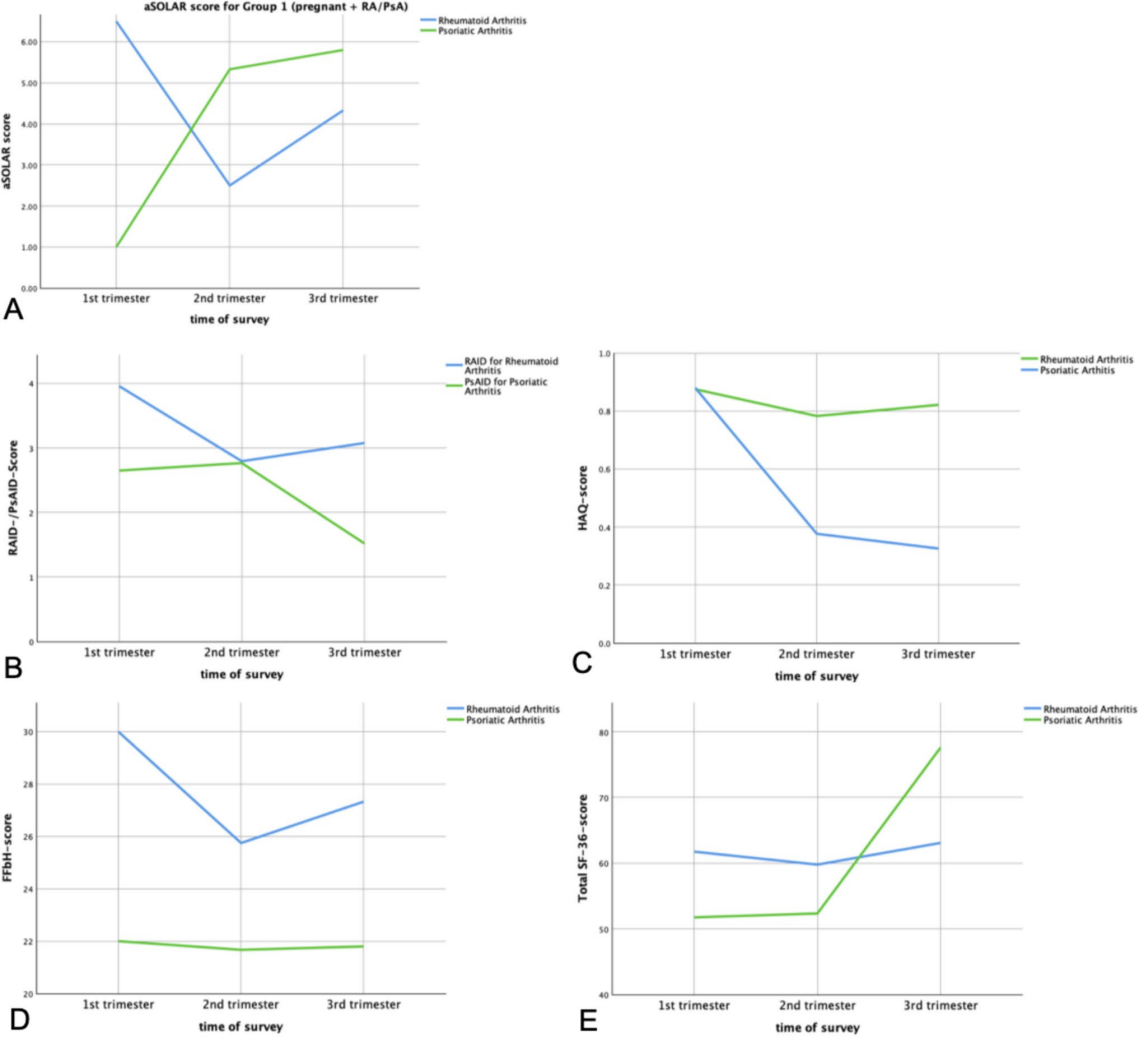
Results of the disease assessments for Group 1 (pregnant + RA/PsA): **a** Mean aSOLAR scores. **b** Mean RAID/PsAID-score in each trimester throughout pregnancy. **c** Mean HAQ-score in each trimester throughout pregnancy. **d** Mean FFbH-score in each trimester throughout pregnancy. **e** Mean SF-36-score in each trimester throughout pregnancy. Abbrv.: aSOLAR: Advanced Sonography of Large Joints in Rheumatology Score, RAID: Rheumatoid Arthritis Impact of Disease, PsAID: 12-item Psoriatic Arthritis Impact of Disease, HAQ: Health Assessment Questionnaire, FFbH: Funktionsfragebogen Hannover, SF-36: 36-Item Short Form Health Survey

Results from self-reported questionnaires on health perception revealed higher scores during the course of pregnancy. Thus, the HAQ and FFbH questionnaires indicated progressively lower mean scores for both pregnant RA and PsA patients (Fig. 4). Comparing pregnant RA and PsA patients to their non-pregnant counterparts, self-reported health perception was predominantly better in pregnant women. Detailed results of disease activity and health perception assessments, including comparisons to pregnant RA and PsA patients and healthy controls, are depicted in Table 2.

### Musculoskeletal ultrasound examination

Pregnant RA patients (Group 1) exhibited lower mean aSOLAR scores over time, although the disease activity for all pregnant RA patients was consistently classified as ‘not active’ by their rheumatologists. In contrast, non-pregnant RA patients (Group 2) had higher mean aSOLAR scores than their pregnant counterparts in the second and third trimesters. One RA patient in Group 2 was classified as having ‘active’ disease activity by their rheumatologist.

Interestingly, in pregnant PsA patients (Group 1), the mean aSOLAR score showed continuously higher results, yet their disease activity was consistently classified as ‘not active’ by the rheumatologist throughout all trimesters. Moreover, non-pregnant PsA patients, despite having more ‘active’ disease cases (with 14.3% classified as having ‘active’ disease), had lower aSOLAR scores compared to pregnant PsA patients in the second and third trimesters. Table 2 displays the results of the mean aSOLAR scores for all groups. Figure 4 illustrates the trajectory of aSOLAR scores in pregnant RA and PsA patients.

## Discussion

To the best of our knowledge, this is the first study to investigate the influence of pregnancy on disease activity in patients with RA and PsA throughout the course of pregnancy using a standardized MSUS protocol. Additionally, our study aimed to evaluate the diagnostic utility of MSUS in assessing disease activity during pregnancy.

In both RA and PsA pregnant women, our study documented improved self-reported measures of functional impairment and disease activity throughout the progression of pregnancy. Assessments using the HAQ, FFbH, SF-36, RAID, and PsAID questionnaires consistently show results indicating less limitations in physical functioning during the progression of pregnancy for both diseases. Although prior research has assessed disease activity levels in RA or PsA during pregnancy using various scores and questionnaires [18, 47, 48], direct comparison with our study is challenging. Nevertheless, our findings align with the majority of existing studies, suggesting that patients with RA or PsA experience enhanced functional status during pregnancy [34], increased physical functionality as well as reduced pain as pregnancy progresses [49] as a result of beneficial changes in disease activity [21, 23]. A meta-analysis reviewing ten studies on the course of rheumatoid arthritis during pregnancy found that approximately 60% of patients experience an improvement in disease activity during gestation [19]. The included studies assessed disease activity using various scoring systems, such as the DAS28, RA Disease Activity Index, the Camp Index, and other composite measures incorporating joint counts and grip strength.

However, self-reported measures of functional impairment and disease activity are subject to potential confounding factors such as bias from the physiological changes accompanying pregnancy. In light of this, our research finds that MSUS may be an alternative, objective diagnostic method for tracking the disease activity in pregnant patients.

Thus, a case report by Kuppers et al. [15] described the first published monitoring disease activity in a pregnant RA patient using MSUS, highlighting its diagnostic potential and advantages. Ultrasound enables the detection of active inflammatory changes, such as synovitis, tenosynovitis, and enthesitis. Several studies have demonstrated that MSUS offers detection rates for synovial inflammation comparable to those of MRI [44, 45, 47, 48]. The use of portable devices like the Butterfly iQ further enhances the clinical applicability of ultrasound, offering a safe, cost-effective, and efficient point-of-care diagnostic tool. Its compact design allows for easy use in outpatient settings and facilitates the detection of findings, such as joint effusion and synovial hyperperfusion [43]. The early detection and close monitoring of inflammatory changes are crucial to prevent irreversible joint damage and long-term functional impairment [15].

When compared to non-pregnant controls, the aSOLAR score among pregnant RA patients shows consistently lower results. Additionally, the aSOLAR scores exhibited continuously lower scores in each trimester over the progression of pregnancy among RA patients in Group 1 indicating less signs of joint inflammation and lower disease activity. These results support previous research [18, 19], suggesting that pregnancy may have a protective effect against RA. The findings suggest that MSUS may be a sensitive and reliable method for tracking disease activity in pregnant RA patients, providing important clinical insights beyond what subjective symptom reporting alone can offer. Immunological and pathophysiological changes during pregnancy, including a Th1-to-Th2 shift, reduced NK cell activity [50–52], and functional alterations in γδT cells, promote feto-maternal tolerance and are associated with improved RA disease activity [53]. This immunomodulatory effect is further supported by hormonal and cytokine changes, while postpartum immune reactivation may contribute to disease flares [19, 54–56].

While our data for pregnant RA patients align well with the existing hypotheses that arthritic disease activity stabilizes or diminishes during pregnancy, we observed the opposite in PsA patients. Contrary to previous studies [20, 21], a rise in aSOLAR scores among pregnant PsA patients suggested higher disease activity toward the end of pregnancy. Since no research has yet examined the immunological basis for variations in PsA activity during pregnancy, this disparity raises questions. It has been suggested that immunomodulatory hormone effects play a key role in psoriasis, with the estrogen–progesterone ratio determining disease activity [22]. While similar cell-associated immunomodulation as seen in RA could be imagined, there is an urgent need for future research focusing on longitudinal disease tracking and concurrent immunological profiling in pregnant PsA patients.

It is interesting to note that self-reported measures of functional impairment and disease activity in PsA patients do not match the results of joint disease activity. This disparity emphasizes how subjective patient perception, especially during pregnancy, may not accurately reflect disease activity, advocating the need for MSUS as an objective imaging indicator for disease activity in the diagnostic protocol. MSUS may improve the assessment of disease status and joint inflammation, allowing the early detection of active synovitis and ultimately improving pregnancy outcomes through early intervention. This is in line with the generally acknowledged idea that effective early treatment during a disease flare can positively alter long-term disease outcomes [57, 58]. The findings demonstrate the feasibility of using a portable device for standardized MSUS assessment, including pregnant populations, supporting its broader implementation in rheumatologic care.

Despite the diagnostic implications of our investigation, it is important to recognize a number of limitations. We acknowledge that the strength of our conclusions is limited by the small sample size with no prior power calculation, as the study was designed as a prospective, observational, and hypothesis-generating investigation. Due to the rare combination of pregnant patients and chronic inflammatory joint disease, our work can only offer preliminary insights into the course of disease activity in pregnant RA and PsA patients tracked by MSUS.

Additionally, while CRP was the primary inflammatory marker assessed in our study, we recognize that including additional laboratory parameters such as ESR could have enriched the analysis. This highlights the broader need for future studies to incorporate extended biomarker panels to enable a more comprehensive evaluation of disease activity during pregnancy.

Larger multi-center investigations are necessary to validate these findings and improve their generalizability to a larger patient population. In this context, a comparison of handheld MSUS and conventional MSUS should be considered. Moreover, correlation analyses should be conducted to evaluate the statistical significance of the findings while accounting for confounding variables such as BMI and age, which varied between the study groups. To clarify the course of disease activity in these patients after delivery and to gain deeper insight into the immunological consequences of pregnancy-related changes, longitudinal studies extending into the postpartum period are required. In our study, however, postpartum follow-up was not feasible, as the majority of participants were referred to our center for pregnancy care and lived at a considerable distance, limiting the possibility of postnatal assessments. Finally, it would be of interest to expand the third control group (healthy pregnant women) by including assessments in the first and second trimesters to analyze the physiological dynamics of joint inflammation and perceived health in healthy pregnancies.

In conclusion, this exploratory study employs MSUS as a diagnostic tool to enhance our understanding of how pregnancy affects disease activity in RA and PsA. While our findings suggest the utility of imaging techniques over self-reported assessments, the results should be interpreted within the limitations of a hypothesis-generating, observational design. Future studies are needed to confirm these initial findings and determine their clinical relevance.

## Data Availability

No datasets were generated or analyzed during the current study.
